# An Experimental Investigation of Viscoelastic Flow in a Contraction Channel

**DOI:** 10.3390/polym13111876

**Published:** 2021-06-04

**Authors:** Wei Wang, Linlin Wang

**Affiliations:** Key Laboratory of Rubber-Plastics, Ministry of Education/Shandong Provincial Key Laboratory of Rubber-Plastics, Qingdao University of Science and Technology, Qingdao 266042, China; qustwllll@163.com

**Keywords:** numerical simulation, complex flow, viscoelastic constitutive models, rheological behavior, polymer melts

## Abstract

In order to assess the predictive capability of the S–MDCPP model, which may describe the viscoelastic behavior of the low-density polyethylene melts, a planar contraction flow benchmark problem is calculated in this investigation. A pressure-stabilized iterative fractional step algorithm based on the finite increment calculus (FIC) method is adopted to overcome oscillations of the pressure field due to the incompressibility of fluids. The discrete elastic viscous stress splitting (DEVSS) technique in combination with the streamline upwind Petrov-Galerkin (SUPG) method are employed to calculate the viscoelastic flow. The equal low-order finite elements interpolation approximations for velocity-pressure-stress variables can be applied to calculate the viscoelastic contraction flows for LDPE melts. The predicted velocities agree well with the experimental results of particle imagine velocity (PIV) method, and the pattern of principal stress difference calculated by the S-MDCPP model has good agreement with the results measured by the flow induced birefringence (FIB) device. Numerical and experimental results show that the S-MDCPP model is capable of accurately capturing the rheological behaviors of branched polymers in complex flow.

## 1. Introduction

During the past two decades with the developments of numerical algorithm and constitutive model, computational rheology has achieved great progress as an important tool. Many investigators have utilized computational rheology to obtain insight into the complex rheological behaviors of polymers, so as to deeply understand the flow status [1] and processing mechanisms, such as extrusion swell [2,3], flow instabilities [4] and secondary flow.

In order to explore the complex behavior of viscoelastic flow, the predictive capability of the constitutive model is of significance for numerical simulation. As for polymer melts, many rheologists put forward several constitutive models, such as the K-BKZ model, PTT model, and Gieseku model. Notably, for better understanding the rheological behaviors of branched polymer melts, McLeish and Larson [5] first proposed the Pom-Pom model that originated from tube theory [6]. The main contribution is to describe the rheological responses of macromolecules in terms of two independent relaxation timescales for orientation and stretch, respectively. After that, several researchers [7,8,9,10,11] developed the Pom-Pom model to remove some defects, so as to reproduce the real rheological behaviors of branched polymer melts. With the help of these constitutive models, we can acquire the orientation and stretch behaviors and reveal the viscoelastic flow mechanism. To validate the numerical results and assess the performance of the constitutive model, some experiments [12,13] are performed. Verbeeten et al. [14,15] calculated viscoelastic flows using the XPP model and obtained good agreement with the experimental results. Wang et al. [16] evaluated the performance of the double-convected Pom-Pom mode (DCPP) model using the particle imagine velocity (PIV) method. They observed that the LDPE melt appears as large corner vortex in the contraction entrance, which has good agreement with the numerical results predicted by Polyflow software. However, they gained less satisfactory for HDPE melt using the same method. 

Numerous studies [1,13,15,17] were carried out both experimentally and numerically for contraction flow, which is a benchmark problem in computational rheology, because there exists a complex combination flow of both shear flow near the channel wall and extension flow along the centerline in the contraction channel. In practice, similar contraction flows also widely appear in industrial polymer processing, such as extrusion flow, injection molding, fiber spinning process, etc. By means of this complex flow field, the rheological responses of the constitutive model may be computed in a numerical way and verified by stress and velocity fields using the birefringence experiment and PIV measurement respectively. Aguayo et al. [18,19] calculated a 4:1 planar rounded-corner and sharp contraction flows governed by the SXPP model and indicated the influence of the number of dangling arms at the end of the pom-pom molecule on the flow behaviors with the aid of the hybrid finite-element/volume scheme. Clemeur et al. [13] exhibited the good performance of the DCPP model with the simulation and experiment results of contraction flow. 

In order to further reveal the rheological behavior of the S-MDCPP model [10], we performed experiments using the 4:1 contraction flow channel for LDPE melt and carried out a numerical study for the extrusion flow. Moreover, to verify the numerical results, the PIV system and flow induced birefringence (FIB) device respectively are utilized to measure the velocity and the principal stress difference of the flow field in this work.

The objective of this investigation is to evaluate the predictive capability of the S-MDCPP model. The 4:1 planar abrupt contraction flow is studied numerically and experimentally, respectively. This paper is organized as follows: Section 2 introduces the material features and experimental set-up. Furthermore, the computational method and model description are also briefly depicted in Section 2. The computational and experimental results together with discussion are described in Section 3. The conclusions of this investigation are drawn in Section 4.

## 2. Materials and Methods

### 2.1. Materials

Low-density polyethylene (DSM Stamylan LD 2008 XC43) was used in the present study. The experimental temperature of the LDPE was 170 °C. See Table 1 for the main properties of the LDPE melt. The shear and uniaxial extension viscosities behaviors of the LDPE melts with various shear rates and tensile rates were depicted in our previous study [20]. The viscoelastic rheological behavior of the LDPE melts is described by the S-MDCPP model [10]. Hence, the model parameters of the S-MDCPP model are solely listed in Table 2 [20].

### 2.2. Experimental Set-Up

(1) Flow Induced Birefringence (FIB) System

**Figure 1:**
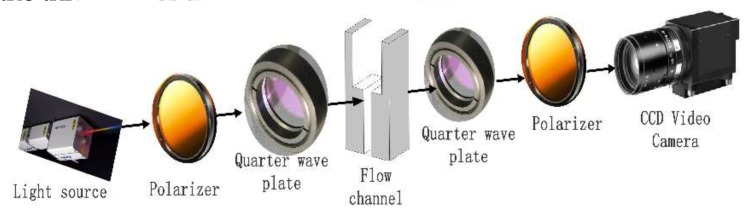
Schematic illustration of the FIB system.

**Figure 2:**
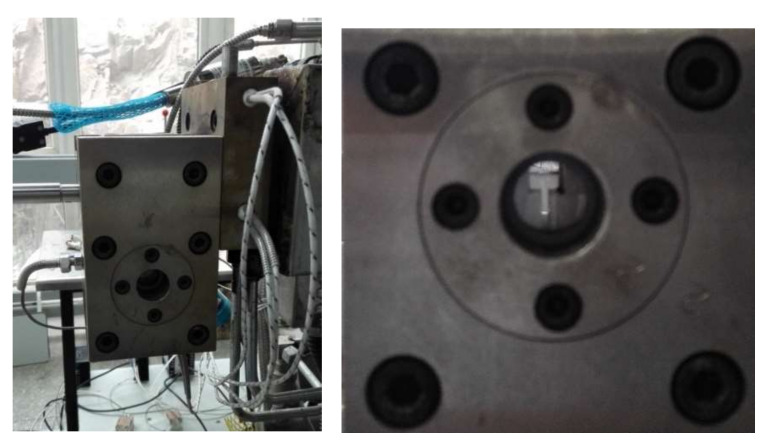
Flow channel set-up.

The optics measurement system consists of two line polarizers, two quarter wave polymer retarders and a high-speed CCD camera. The schematic illustration of the FIB system is depicted in Figure 1. A laser device made by Beijing Laserwave Optoelectronics Technology Co., Ltd. is utilized to produce green light of wavelength λ = 532 nm. The flow channel device shown in Figure 2 has a width of 8 mm and height of 45 mm in the upstream channel and width of 2 mm and height of 45 mm in the downstream channel, and the thickness of the whole flow channel is 100 mm.

(2) Particle imagine velocity (PIV) system

The PIV measurement system made by Beijing Cubetiandi Science and Technology Development Co. Ltd. (Beijing, China) is used to obtain the velocities of the contraction flow field. The PIV system composes of a high-speed CCD camera, a laser with green light of wavelength λ = 532 nm and imaging analysis software.

### 2.3. Computational Method and Model Description

In this investigation, the pressure stabilized iterative fractional step algorithm adopted in our previous studies [10,20,21,22] will be applied to calculate the LDPE melts flow behavior in the contraction channel. It is worth noting that the finite increment calculus (FIC) method [23,24] is employed to reformulate the stabilized form of mass conservation equation to achieve the stable solution of the pressure field. Accordingly, the equal low-order triangle elements may be applied to discretize the flow field. Meanwhile, the discrete elastic viscous stress splitting (DEVSS) method of Guénette and Fortin [25] in combination with the streamline upwind Petrov-Galerkin (SUPG) approach [26] is applied to calculate the viscoelastic flow. The computational method is described more fully in our previous studies [10,20,21].

**Figure 3:**
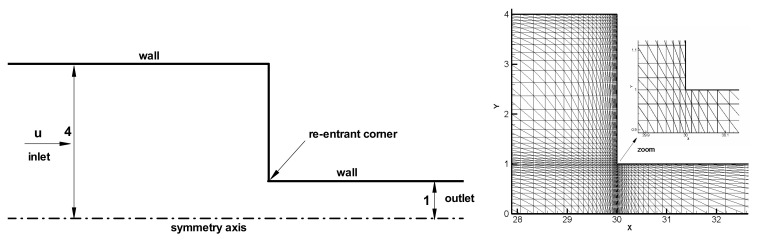
Schematic and computational mesh around the re-entrant corner of 4:1 planar contraction.

In the LDPE melt extrusion process, when the melt leaves the extruder die, it is squeezed into a home-made flow channel with two glass windows, as shown in Figure 2. The flow device consists of three plates, which create a 4:1 contraction channel. Because the ratio of thickness (depth) to width is above 10, the three-dimensional channel may be simplified to a planar model. So, a planar 4:1 contraction geometry is constructed in this study for calculating the viscoelastic flow. Because the contraction flow as a benchmark problem [1,13,15,17] is usually used to evaluate the capability of constitutive models and numerical algorithms. Accordingly, the planar 4:1 contraction viscoelastic flow is also calculated in this study. The finite element mesh model is displayed in Figure 3. The velocities at entrance and exit are prescribed by a fully developed velocity profile, respectively. The average velocity of the entrance is 1.275 mm/s. The vertical velocity at the centerline of the channel is set as zero. The no-slip boundary conditions are considered on the channel wall. The pressure of the symmetric point at the exit is specified zero. All components of the viscoelastic extra stress are initially applied by the computational results achieved from the UCM fluid which possesses the same Maxwell parameters as those used for the S–MDCPP model.

## 3. Results and Discussion

**Figure 4:**
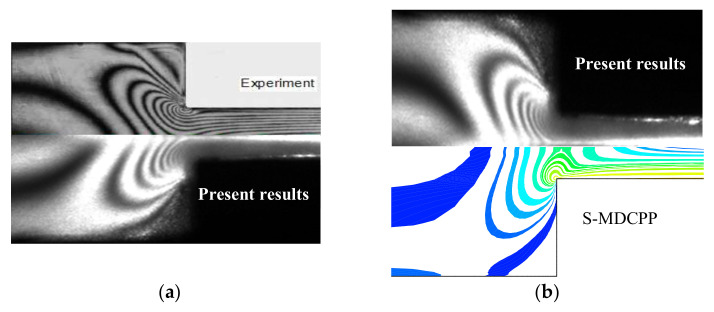
Comparison of the isochromatic fringe patterns measured by Verbeeten [27] and our experimental result (**a**), our experimental and computational results (**b**).

Figure 4 shows the sochromatic fringe patterns of the principal stress difference (PSD), that is,$\;\mathrm{PSD}=\sqrt{{\left(\tau_{xx}-\tau_{yy}\right)}^{2}+4\tau_{xy}^{2}}$. It can be seen that our experimental result agrees well with the PSD patterns measured by Verbeeten [15,27]. Because the channel contraction ratio of the experimental device used by Verbeeten is 3.29:1, but the contraction ratio of this study is 4:1, the size of the PSD pattern has a slight difference in the contraction region. However, the number and location of the PSD field pattern are consistent with the results reported by Verbeeten. Moreover, the computational results using our calculation scheme have good quantitative agreement with the measured PSD pattern by means of our FIB device, so the stability and accuracy of the computational method adopted in this study are verified. By comparison between numerical and experimental results, we observe the S-MDCPP model has good predictive capability and may reproduce the complex rheological behavior of branched polymer melts. The vortex behavior and butterfly pattern of PSD are well-predicted by the S-MDCPP model, which is the typical features of LDPE melts flow through the contraction channel. During the branched polymer melts flow into the contraction entry, the stronger shear stress occurs near the channel wall, while the higher extensional behavior appears along the centerline, which results in high extensional viscosity. These complex flow behaviors induce shear thinning and extension thickening (or strain hardening) of branched macromolecules occurring simultaneously in the contraction channel, which demonstrates the complicated butterfly pattern of PSD.

**Figure 5:**
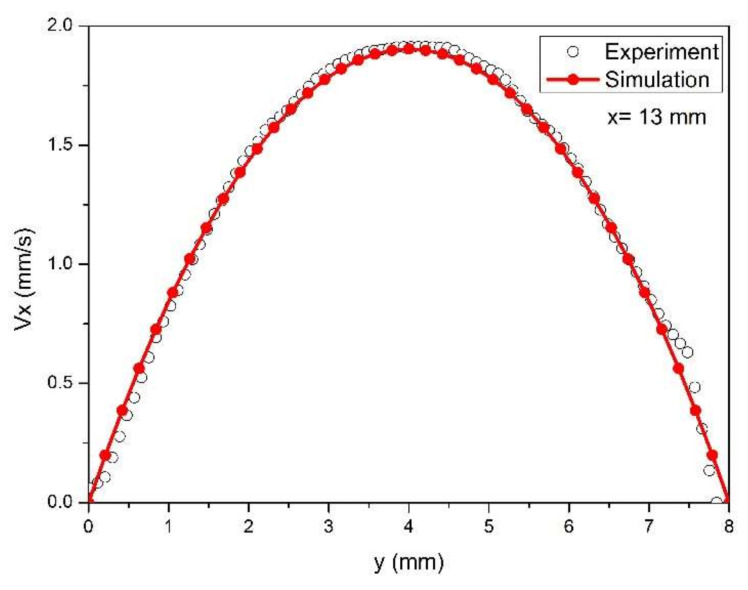
Comparison of the PIV measured velocity and predicted velocity in the upstream cross section of x = 13 mm.

Figure 5 displays the horizontal velocity of the upstream channel in the cross section of x = 13 mm. It can be seen that the predicted velocities agree very well with the results measured by the PIV method except for the neighborhood of the channel wall. This indicates the pressure stabilized iterative fractional step algorithm adopted in this study is reliable, although the equal low-order triangle elements are applied to calculate the velocity field. The distributions of sectional velocities display a parabolic curve. 

**Figure 6:**
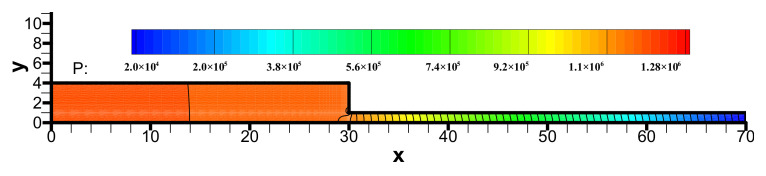
Distribution of pressure in the flow field.

The pressure distribution in the viscoelastic contraction flow is shown in Figure 6. We can see the pressure field is stable, although the equal low-order triangle elements are applied to calculate the pressure, velocity, viscoelastic extra-stress tensor and discrete rate-of-strain tensor. This good stability feature attributes to the FIC approach introduced a pressure stabilization term. 

**Figure 7:**
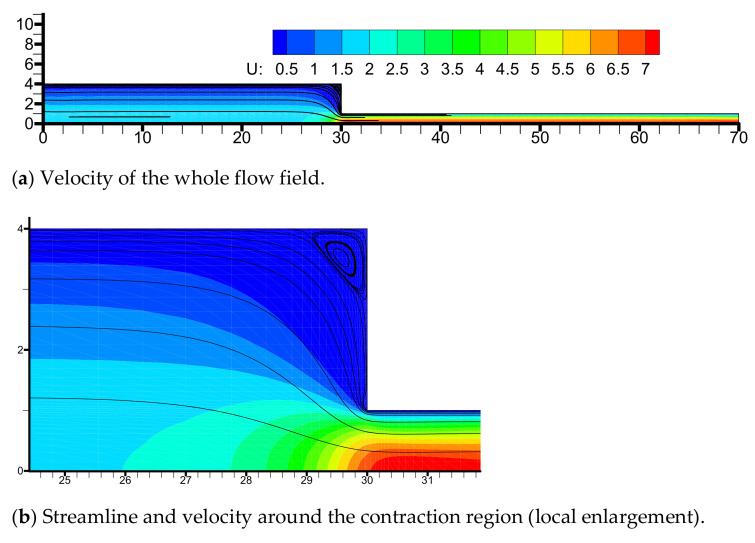
Distributions of horizontal velocity (**a**) and streamline (**b**) in the flow field.

The horizontal velocity and streamline are exhibited in Figure 7. We note that the horizontal velocity near the centerline of the downstream channel is larger than other locations. Moreover, we observe a large rotating vortex appears in the salient corner of the upstream channel (See Figure 7b), which is the typical feature of the LDPE melts flowing through a contraction channel. The corner vortex behavior is in agreement with the results of several researchers [1,13,15].

**Figure 8:**
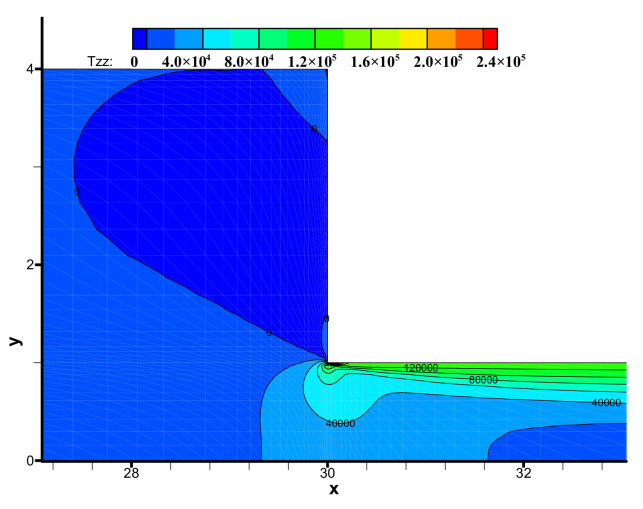
Distribution of stress τ_xx_ in the flow field near the contraction region.

**Figure 9:**
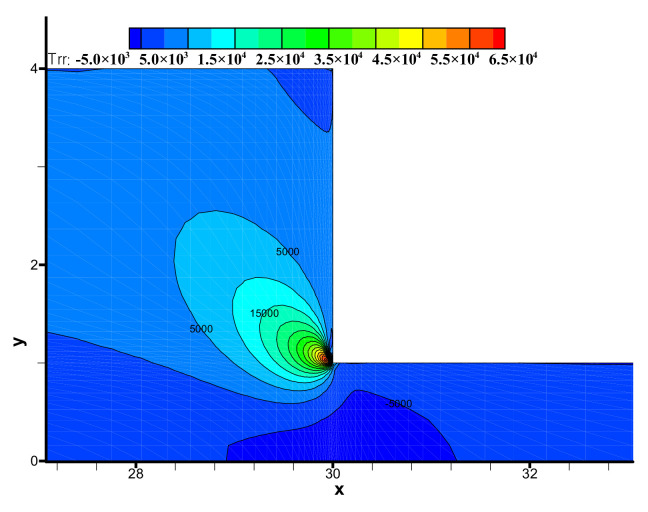
Distribution of stress τ_yy_ in the flow field near the contraction region.

**Figure 10:**
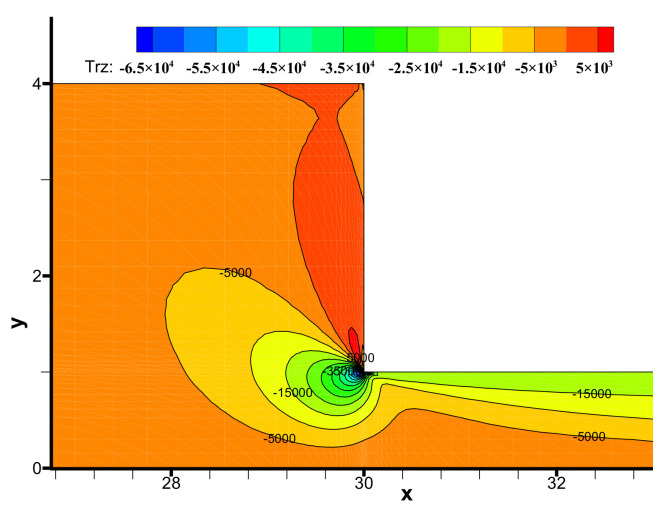
Distribution of stress τ_xy_ in the flow field near the contraction region.

The components (i.e., τ_xx_, τ_yy_, τ_xy_) of the viscoelastic extra-stress tensor are shown in Figure 8, Figure 9 and Figure 10, respectively. We may observe the viscoelastic extra-stress field is stable. The largest magnitude of the stresses is located in the sharp re-entrant corner. In addition, the stresses of τ_xx_ and τ_xy_ are larger in the vicinity of the solid wall, where the shear rates are higher than other regions of the channel.

**Figure 11:**
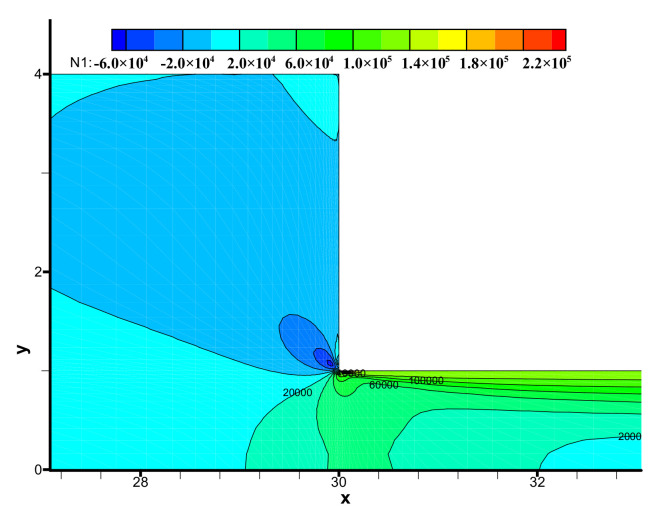
Distribution of the first normal stress difference *N*_1_ in the flow field near the contraction region.

The distribution of first normal stress difference *N*_1_ ($N_{1}=\tau_{xx}-\tau_{yy}$) is shown in Figure 11. It is observed that the distribution of *N*_1_ near the sharp corner is complex. Meanwhile, the largest first normal stress difference is located in this corner.

**Figure 12:**
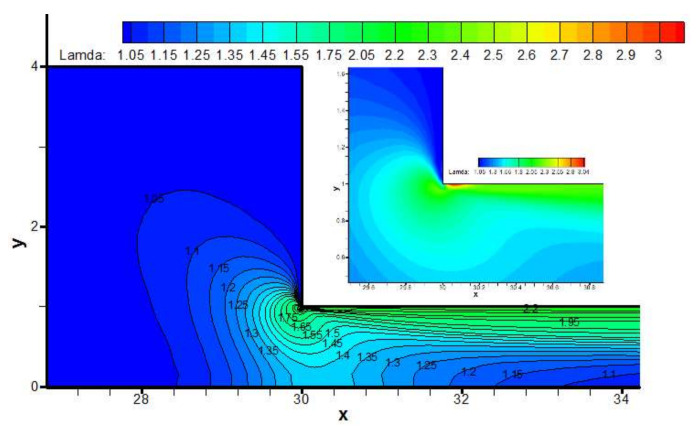
Distribution of backbone stretch Λ in the flow field near the contraction region.

**Figure 13:**
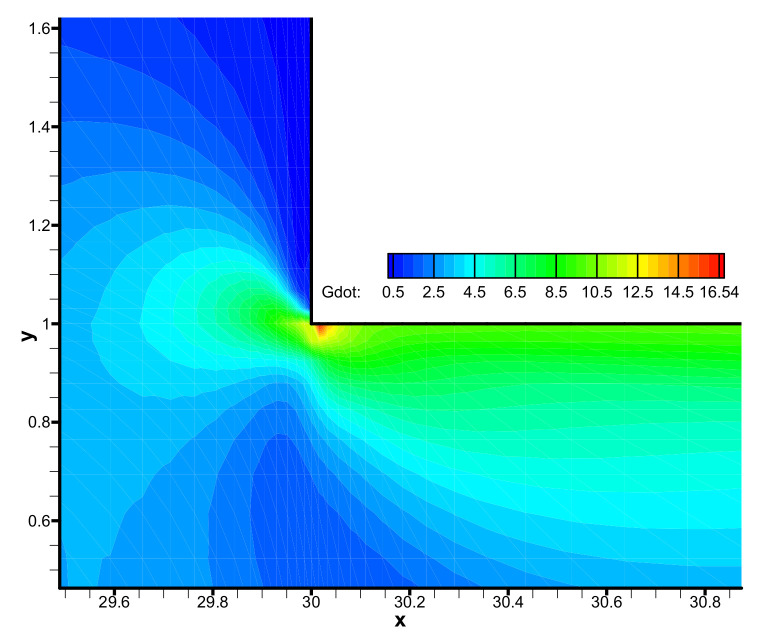
Distribution of shear rate in the flow field near the contraction corner.

The backbone stretch distribution of the branched macromolecules is shown in Figure 12. We may observe the larger backbone stretch locates in the vicinity of the contraction sharp corner (See the inset in Figure 12), and the largest backbone stretch of 3.04 occurs in the neighborhood of the re-entrant corner near the downstream wall, where there is the maximum shear rate, as shown in Figure 13. The very high shear rate induces large stretch orientation of the macromolecules. 

**Figure 14:**
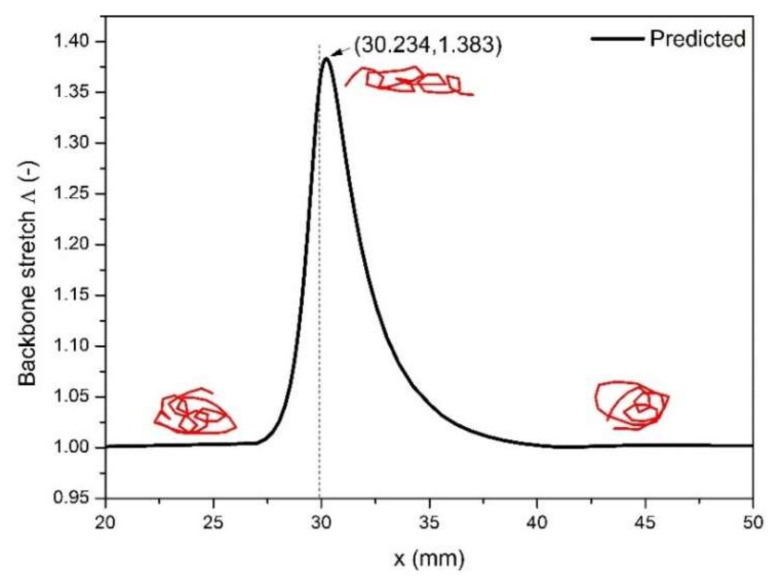
Distribution of backbone stretch along the axis of symmetry.

From Figure 14, we can see the backbone stretch closes approximately to a value of 1, when macromolecules reach far away from the contraction entrance wherever the upstream or downstream. However, when macromolecules flow through the contraction channel, because the larger axial velocity induces stronger orientation of the macromolecule, the larger backbone stretch appears in the vicinity of the contraction entrance. After that, the macromolecules begin to relax toward recovering the random coil status.

## 4. Conclusions

The viscoelastic flow of branched polymer melts (LDPE) in a 4:1 contraction flow channel was studied by using the FIB and PIV experiment devices, along with the pressured stabilized iterative fractional step algorithm. Firstly, the numerical results demonstrate that the S-MDCPP model is capable of reproducing the rheological behaviors of the branched polymers in the complex flow. Secondly, the results show that the predicted PSD pattern agrees well with the experimental results obtained by the FIB device, and the velocities calculated by the numerical scheme have good agreement with the PIV measured results. Consequently, the viscoelastic constitutive model (e.g., S-MDCPP model) originated from tube theory may capture the complex rheological behaviors of polymers. From the point of numerical result about the stretch behavior, the larger backbone stretch locates in the vicinity of the contract sharp corner, and the largest backbone stretch occurs in the neighborhood of the re-entrant corner near the downstream wall, where the maximum shear rate occurs, mainly because the high shear rate induces a large stretch orientation of the macromolecules. Based on the above results, we consider that the S-MDCPP model may be a good candidate for complex viscoelastic flow simulation in extrusion and injection molding in the future. The reliable and accurate numerical prediction may help us to reveal and deeply understand the complex rheological behaviors of polymers.

## Figures and Tables

**Table 1:** Materials properties of the experiment.

	Density (kg/m^3^)	Mw	Mw/Mn	Melt Index (dg/min)
LDPE (Stamylan LD 2008 XC43)	920	155,000	11.92	8

**Table 2:** Maxwell and nonlinear parameters of DSM Stamylan LD 2008 XC43 LDPE melt [20].

S–MDCPP Model					
**Mode**	Maxwell Parameters		Nonlinear Parameters		
	G (Pa)	λ_0b_ (s)	*q* = 2/ν	*r*	ξ
1	1.0377 × 10^4^	0.40	10	1.2	0.02

Note that *q* represents the number of arms at the end of a backbone, *r* equals λ_0b_/λ_s_, where λ_0b_ and λ_s_ denote orientation and stretch relaxation time, respectively, and ξ denotes the slip coefficient, which introduces the non-zero second normal stress difference as adopted in the DCPP model. Moreover, ν reflects the influence of the neighborhood polymer chains on the backbone tube stretch. In Table 2, *r* and ξ are dimensionless parameters.

## Data Availability

Data is contained within the article or Supplementary Materials.

## Supplementary Materials

The following are available online at https://www.mdpi.com/article/10.3390/polym13111876/s1, Table S1: Shear viscosities under different shear rates, Table S2: Uniaxial viscosities under different ex-tension rates.

## References

[1] Hooshyar S., Germann N. (2019). Shear banding in 4: 1 planar contraction. Polymers.

[2] Hooshyar S., Germann N. (2019). The investigation of shear banding polymer solutions in die extrusion geometry. J. Non-Newton. Fluid Mech..

[3] Ganvir V., Gautham B.P., Pol H., Bhamla M.S., Sclesi L., Thaokar R., Lele A., Mackley M. (2011). Extrudate swell of linear and branched polyethylenes: ALE simulations and comparison with experiments. J. Non-Newton. Fluid Mech..

[4] Pol H.V., Joshi Y.M., Tapadia P.S., Lele A.K., Mashelkar R.A. (2007). A Geometrical Solution to the Sharkskin Instability. Ind. Eng. Chem. Res..

[5] McLeish T.C.B., Larson R.G. (1998). Molecular constitutive equations for a class of branched polymers: The pom-pom polymer. J. Rheol..

[6] McLeish T.C.B. (2002). Tube theory of entangled polymer dynamics. Adv. Phys..

[7] Verbeeten W.M.H., Peters G.W.M., Baaijens F.P.T. (2001). Differential constitutive equations for polymer melts: The extended Pom-Pom model. J. Rheol..

[8] Clemeur N., Rutgers R.P.G., Debbaut B. (2003). On the evaluation of some differential formulations for the Pom-Pom constitutive model. Rheol. Acta..

[9] Tanner R.I., Nasseri S. (2003). Simple constitutive models for linear and branched polymers. J. Non-Newton. Fluid Mech..

[10] Wang W., Li X.K., Han X.H. (2010). A numerical study of constitutive models endowed with Pom-Pom molecular attributes. J. Non-Newton. Fluid Mech..

[11] Clemeur N., Debbaut B. (2007). A pragmatic approach for deriving constitutive equations endowed with pom–pom attributes. Rheol. Acta..

[12] Marín-Santibáñez B.M., Pérez-González J., Gómez-Herrera G., Rodríguez-González F. (2020). Capillary extrusion of polypropylene/high-density polyethylene immiscible blends as studied by rheo-particle image velocimetry. Polym. Test..

[13] Clemeur N., Rutgers R.P.G., Debbaut B. (2004). Numerical simulation of abrupt contraction flows using the Double Convected Pom–Pom model. J. Non-Newton. Fluid Mech..

[14] Verbeeten W.M.H., Peters G.W.M., Baaijens F.P.T. (2002). Viscoelastic Analysis of Complex Polymer Melt Flows using the eXtended Pom-Pom model. J. Non-Newton. Fluid Mech..

[15] Verbeeten W.M.H., Peters G.W.M., Baaijens F.P.T. (2004). Numerical simulations of the planar contraction flow for a polyethylene melt using the XPP model. J. Non-Newton. Fluid Mech..

[16] Wang X.L., Chen R.H., Wang M.M., Jin G. (2015). Validation of Double Convected Pom-Pom Model With Particle Image Velocimetry Technique. Polym. Eng. Sci..

[17] Owens R.G., Phillips T.N. (2002). Computational Rheology.

[18] Aguayo J.P., Tamaddon-Jahromi H.R., Webster M.F. (2006). Extensional response of the pom-pom model through planar contraction flows for branched polymer melts. J. Non-Newton. Fluid Mech..

[19] Aguayo J.P., Phillips P.M., Phillips T.N., Tamaddon-Jahromi H.R., Snigerev B.A., Webster M.F. (2007). The numerical prediction of planar viscoelastic contraction flows using the pom-pom model and higher-order finite volume schemes. J. Comput. Phys..

[20] Wang W., Wang X.P., Hu C.X. (2014). A comparative study of viscoelastic planar contraction flow for polymer melts using molecular constitutive models. Korea-Aust. Rheol. J..

[21] Wang W., Hu C.X., Li W.W. (2016). Time-dependent rheological behavior of branched polymer melts in extensional flows. Mech. Time-Depend. Mater..

[22] Li X.K., Han X.H. (2010). Numerical modeling of viscoelastic flows using equal low-order finite elements. Comput. Meth. Appl. Mech. Engrg..

[23] Oñate E. (2000). A stabilized finite element method for incompressible viscous flows using a finite increment calculus formulation. Comput. Meth. Appl. Mech. Engrg..

[24] Li X.K., Han X.H. (2005). An iterative stabilized fractional step algorithm for numerical solution of incompressible N–S equations. Int. J. Numer. Meth. Fluids..

[25] Guénette R., Fortin M. (1995). A new mixed finite element method for computing viscoelastic flows. J. Non-Newton. Fluid Mech..

[26] Brooks A.N., Hughes T.J.R. (1982). Streamline upwind/Petrov–Galerkin methods for convection dominated flows with particular emphasis on the incompressible Navier–Stokes equations. Comput. Meth. Appl. Mech. Engrg..

[27] Verbeeten W.M.H. (2001). Computational Polymer Melt Rheology. Ph.D. Thesis.

