# Supplementary material for: An Experimental Investigation of Viscoelastic Flow in a Contraction Channel
Source: Polymers (Basel). 2021 Jun 4;13(11):1876. doi: 10.3390/polym13111876 (PMC8201106; doi:10.3390/polym13111876)
Supplement: Supplementary file 1 [file polymers-13-01876-s001.zip › polymers-1235034-supplementary.pdf]

The shear and uniaxial extension viscosities of the LDPE (DSM Stamylan LD 2008 XC43) melts with various shear rates and extension rates are listed in Tables 1 and 2. These experimental data may be found on Page 108 in Verbeeten's Ph.D thesis. (Verbeeten, W.M.H. Computational Polymer Melt Rheology. Ph.D. Thesis, Technische Universiteit Eindhoven, Eindhoven, Netherlands, 2001.)

Table 1 Shear viscosities under different shear rates

Shear rate (1/s)	Shear viscosity (Pa.s)
0.0304	4180
0.0604	4070
0.151	3860
0.354	3560
0.901	2920
1.5	2840
2.02	2560
3.55	2130
5.51	1840
6.96	1910
9.16	1530
15	1220
17.2	1250
40.3	831
80.1	559
170	352
421	218
908	153

Table 2 Uniaxial viscosities under different extension rates

Extension rate (1/s)	Uniaxial viscosity (Pa.s)
0.38259	55530
1.1045	80373
3.763	67939
